# Microbicides Development Programme: Engaging the community in the standard of care debate in a vaginal microbicide trial in Mwanza, Tanzania

**DOI:** 10.1186/1472-6939-10-17

**Published:** 2009-10-09

**Authors:** Andrew Vallely, Charles Shagi, Shelley Lees, Katherine Shapiro, Joseph Masanja, Lawi Nikolau, Johari Kazimoto, Selephina Soteli, Claire Moffat, John Changalucha, Sheena McCormack, Richard J Hayes

**Affiliations:** 1 London School of Hygiene & Tropical Medicine, Keppel Street, London WC1E 7HT, UK; 2 African Medical and Research Foundation, PO Box 1482, Mwanza, Tanzania; 3 National Institute for Medical Research, PO Box 1462, Mwanza, Tanzania; 4 Consultant, Global Campaign for Microbicides/PATH, Middlesex, Vermont, USA; 5 Stakeholders Advisory Group, Microbicides Development Programme, Mwanza, Tanzania; 6 Community Advisory Committee, Microbicides Development Programme, Mwanza, Tanzania; 7 Medical Research Council Clinical Trials Unit, 222 Euston Road, London NW1 2DA, UK

## Abstract

**Background:**

HIV prevention research in resource-limited countries is associated with a variety of ethical dilemmas. Key amongst these is the question of what constitutes an appropriate standard of health care (SoC) for participants in HIV prevention trials. This paper describes a community-focused approach to develop a locally-appropriate SoC in the context of a phase III vaginal microbicide trial in Mwanza City, northwest Tanzania.

**Methods:**

A mobile community-based sexual and reproductive health service for women working as informal food vendors or in traditional and modern bars, restaurants, hotels and guesthouses has been established in 10 city wards. Wards were divided into geographical clusters and community representatives elected at cluster and ward level. A city-level Community Advisory Committee (CAC) with representatives from each ward has been established. Workshops and community meetings at ward and city-level have explored project-related concerns using tools adapted from participatory learning and action techniques e.g. chapati diagrams, pair-wise ranking. Secondary stakeholders representing local public-sector and non-governmental health and social care providers have formed a trial Stakeholders' Advisory Group (SAG), which includes two CAC representatives.

**Results:**

Key recommendations from participatory community workshops, CAC and SAG meetings conducted in the first year of the trial relate to the quality and range of clinic services provided at study clinics as well as broader standard of care issues. Recommendations have included streamlining clinic services to reduce waiting times, expanding services to include the children and spouses of participants and providing care for common local conditions such as malaria. Participants, community representatives and stakeholders felt there was an ethical obligation to ensure effective access to antiretroviral drugs and to provide supportive community-based care for women identified as HIV positive during the trial. This obligation includes ensuring sustainable, post-trial access to these services. Post-trial access to an effective vaginal microbicide was also felt to be a moral imperative.

**Conclusion:**

Participatory methodologies enabled effective partnerships between researchers, participant representatives and community stakeholders to be developed and facilitated local dialogue and consensus on what constitutes a locally-appropriate standard of care in the context of a vaginal microbicide trial in this setting.

**Trial registration:**

Current Controlled Trials ISRCTN64716212

## Background

There has been considerable debate over the last 10-15 years as to what constitutes an appropriate standard of health care (SoC) in the context of clinical trials conducted in resource-poor countries [1-3].

HIV prevention trials present a number of unique ethical dilemmas for researchers [4-12]. In many developed and developing countries, such trials are feasible only among vulnerable sub-populations at high-risk of HIV infection and sexually transmitted infections (STIs)[13,14], where poverty, stigma and social exclusion are significant barriers to local health care access and decisions regarding SoC provision are therefore paramount [15-18].

There is currently no international consensus on researchers' responsibility to meet the health needs of clinical trial participants or their obligations to the wider community from which participants are drawn. In 2000, the World Medical Association (WMA) revised the Declaration of Helsinki, stating in paragraph 29 that the "best current prophylactic, diagnostic and therapeutic methods" i.e. the highest possible SoC should be made available to people participating in clinical research [19]. Guidelines produced subsequently by the UK Nuffield Council on Bioethics [20], the US National Bioethics Advisory Commission (NBAC) [21], the Council for International Organization of Medical Sciences (CIOMS) [22] and UNAIDS [23] contradict this construct of SoC however and propose that it is ethically justifiable, under certain conditions, to provide less than the worldwide best standard. For example, both the NBAC and CIOMS guidelines recommend that clinical trial participants be given "established effective" therapy as a minimum whilst the Nuffield Council on Bioethics recommends:

*"where it is not appropriate to offer a universal *[i.e. 'worldwide best'] *standard of care, the minimum standard of care that should be offered to the control group is the best intervention available for that disease as part of the national public health system"*.

More recently, international debate has shifted from these narrow definitions of SoC, in which researchers' obligation to subjects randomised to the placebo or control arms of HIV prevention trials are a particular focus, to a broader conceptualisation of SoC based on the principles of equity, social justice and beneficence that consider the overall health needs of trial participants and the community within which research is conducted [16,24]. This approach is supported by recent community-based research in East and Southern Africa, India and the United States, which has highlighted the pivotal role of community participation and the importance of open, effective dialogue between researchers and community stakeholders in the on-going SoC debate [12,17,18,25].

Mwanza is one of six centres in sub-Saharan Africa participating in the Microbicides Development Programme (MDP), an international partnership for the development of vaginal microbicides for HIV prevention, funded by the UK Department for International Development and Medical Research Council (MRC)[26]. A feasibility study [27] was carried out among an occupational cohort of women at increased risk of HIV infection and STIs in ten administrative wards in Mwanza City, northwest Tanzania between July 2002 and March 2005 in preparation for the on-going MDP301 randomized placebo-controlled efficacy and safety trial of the candidate vaginal microbicide PRO2000/5 Gel (Indevus Pharmaceuticals, USA), which started in November 2005. Women working in food and recreational facilities, including modern bars, traditional bars (known as *vilabu *or pombe shops in Tanzania), restaurants, hotels, guesthouses, groceries and as informal food vendors (known locally as *mamalishe*), are eligible to participate. Research conducted at a number of sites in Tanzania suggests that some women in this occupational group periodically supplement their income through transactional sex [28,29] and are hence at increased risk of STIs and HIV infection [27,30-32]

In this paper we describe how a locally-appropriate SoC package was developed by researchers, study participants and community stakeholders in the context of the MDP301 vaginal microbicide trial in Mwanza; present case studies to illustrate some of the ethical issues and dilemmas encountered during implementation; and critically appraise whether the strategies adopted have been successful in this setting.

## Methods

### Study population

The design of the microbicide trial feasibility study in Mwanza and the baseline socio-demographic, behavioural and biomedical characteristics of study participants have previously been described [27]. In brief, following participatory community mapping to identify eligible food and recreational facilities in ten administrative wards in Mwanza City, a community-based clinic was established in a guesthouse in each ward by October 2002. Study clinics provided free sexual and reproductive health services to participants including voluntary HIV counselling and testing; STI syndromic management; family planning advice and contraceptive methods; and health education. Participants found to be HIV positive were referred to a specialist local public health provider for clinical assessment and care which included antiretroviral provision if appropriate. In addition, a system of referral to a network of local non-governmental and community-based organizations (NGOs and CBOs) providing care and support for participants and their families living with HIV and AIDS was established. Participants with general medical or gynaecological problems were referred to established local care providers for further clinical assessment. A total of 1573 women were enrolled and followed up at three-monthly intervals for a maximum of two years. The feasibility study ended in March 2005 with the completion of a small pilot study, conducted among 59 participants, to investigate the acceptability of HPTN035 Placebo Gel in this study population and the feasibility and acceptability of proposed clinical trial procedures [33].

Following the successful completion of the feasibility and pilot studies in Mwanza, the MDP301 efficacy and safety trial started in November 2005. A total of 1146 HIV sero-negative subjects have been enrolled into the main trial in Mwanza, with follow-up completed in mid-2009. Trial participants, and women who participated in the feasibility and/or pilot studies, are all eligible to receive free sexual and reproductive health services through the community-based study clinics and have access to ancillary care by referral (Table 1).

**Table 1:** MDP301 Mwanza clinical care package

**Eligibility criteria: summary of those eligible to receive clinical care package provided through study clinics and referral services**
All current MDP301 trial participants (scheduled clinical review as per trial protocol plus drop-in visits at any time)
All women who attended a screening visit for the MDP301 trial but were subsequently excluded or decided not to enrol (drop-in visits at any time)
All women who participated in the MDP Mwanza feasibility or pilot study (drop in visits at any time)
Male sexual partners of any of the above women were eligible for STI referral services

**Summary of care provided in study clinics and via referral services**

*Management of STIs/RTIs*

Syndromic STI management (based on national guidelines produced by the MoH in Tanzania[36]) supplemented by vaginal pH for bacterial vaginosis and collection of genital specimens and venepuncture according to trial Standard Operating Procedures (SOPs)
Clinical management subsequently adjusted as appropriate at next clinic visit based on laboratory test results (*N. gonorrhoeae, C. trachomatis, T. vaginalis, Herpes simplex type-2*, syphilis, bacterial vaginosis)
Sexual partners are advised to attend a designated collaborating local service provider for free STI care (women provided with referral slips; anonymised notification sent to a designated local STI care service provider)

*HIV diagnosis and care*

Voluntary HIV testing and counselling (VCT): same-day service using parallel rapid diagnostic tests supplemented by laboratory-based ELISA confirmation as appropriate and as specified in site SOPs
Women found to be HIV seropositive at the MDP301 screening visit, who seroconvert during the trial or who were diagnosed as HIV seropositive during the feasibility study are referred to a local collaborating specialist centre providing free HIV clinical care and support, including CD4 count estimation, diagnosis and management of tuberculosis and opportunistic infections, antiretroviral drug therapy and clinical monitoring
Counselling and support for women found to be HIV positive is provided at study clinics and via a local referral network established with CBOs and NGOs in Mwanza through the community liaison system. Free specialist support for women living with HIV is also available through this network e.g. legal advice regarding land and housing issues and related permanency arrangements

*Family planning*

Free counselling and advice regarding different forms of family planning are provided in study clinics, which also provide condoms, combined oral contraceptives and Depo-Provera injections as appropriate
Women requesting tubal ligation or intrauterine contraceptive devices (IUCD) are referred to local designated service providers

*Gynaecology, general medical and child health services*

General medical and genital examinations are conducted in all subjects at the MDP301 screening visit. Genital examination is subsequently scheduled at three-monthly intervals in the MDP301 trial protocol. General and genital examinations are also available as indicated at any time
Women found to have a gynaecological abnormality (e.g. suspected carcinoma of the cervix identified macroscopically on speculum examination) are referred to a designated specialist who provides expedited care on a private patient basis. Referral costs are routinely met by the study. Summary medical reports are provided to the study team for filing in patient clinical record folders
Women found to have a general medical condition (e.g. hypertension, diabetes) are referred to one of several local physicians. Referral costs are met by the study as required
Participants requesting services or advice for their children (e.g. for childhood fever or other medical conditions) are advised to attend free local child health clinics for assessment and clinical management and are not treated for malaria or other conditions at MDP Mwanza study clinics

### Community liaison

During the Mwanza feasibility study, a community liaison system (CLS) was established, based on 78 geographical clusters of facilities in ten city wards [34], and has used participatory research tools to facilitate open dialogue and partnership working practices between researchers, study participants and community representatives [25]. The definition of 'community' adopted in Mwanza was conceived and articulated by study participants who perceived themselves not only as part of an occupational group, but as a discrete community with shared social ties, perspectives and experiences within a defined geographical location [34,35]. Cluster, ward and city-level representatives have been elected in a process facilitated by the site Community Liaison Officer (CLO) and a Community Advisory Committee (CAC) established. In community workshops at ward and city level, listing, ranking, diagramming and other techniques were used to capture and prioritise project-related concerns, including issues related to the scope and quality of health services provided by study clinics to participants, their families and the broader community [25]. With the start of the MDP301 trial in Mwanza, the CLS has been consolidated and expanded with new facility clusters and associated representatives developed in close consultation with the community. Participatory community workshops have continued every 4-6 months during the main trial in order to monitor community perceptions of the study and in particular, to ensure that decisions about clinical services provided by or through the project are taken in consultation with 'primary' stakeholders i.e. potential and actual study participants.

### Stakeholders Advisory Group

During the feasibility study, researchers established links at all levels with key local public-sector, non-governmental and community-based stakeholders providing health and social support in Mwanza ('secondary stakeholders'). Senior staff provided six-monthly progress updates to the Mwanza City HIV/AIDS Management Committee, which provided a useful forum in which to discuss a broad range of issues with representatives from the local public and non-governmental sector, including SoC and the ethics of trial participation in vulnerable communities. These meetings led to the formation of a project-specific Stakeholders Advisory Group (SAG) in June 2006, designed to complement the guidance and advice provided through the CAC. During the MDP301 trial, the SAG has continued to meet routinely every 4-6 months to review progress and to make suggestions as to how clinical care for trial participants could be consolidated, improved or delivered in alternative ways. The SAG now includes representatives from the City health department, local public health providers, non-governmental and community-based organisations (NGOs and CBOs) active in HIV care and support and two representatives from the CAC.

### Ethical approval

Ethical clearance for the feasibility study and the MDP301 clinical trial in Mwanza was obtained from the National Medical Research Coordinating Committee in Tanzania and the Ethics Committee of the London School of Hygiene and Tropical Medicine UK. Written informed consent (signature or witnessed thumbprint) is obtained from all participants prior to enrolment.

A SoC statement, encapsulating the clinical care package described in Table 1 above, was ratified by the Ethics Committees in Tanzania and UK and by the Mwanza Community (CAC) and Stakeholders (SAG) Advisory Groups prior to the start of the main trial.

## Results

### Community workshops and CAC meetings

**Figure 1:**
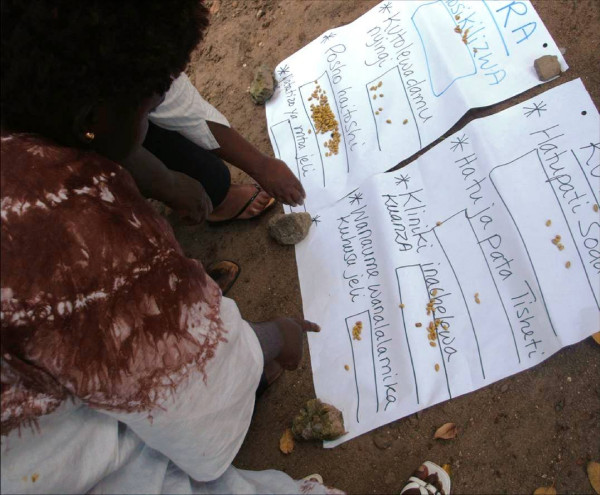
**This figure is a photograph titled Using seeds to rank priorities**.

Community workshops in Mbugani, Pamba, Igoma and Nyamanoro wards conducted between October and December 2006 highlighted a variety of issues related to the scope and range of clinical services provided during the first year of the MDP301 trial in Mwanza that were further investigated during two participatory CAC meetings conducted in January and February 2007. Listing, ranking, diagramming and pairwise matrices were used (Figure 1) to establish a list of priority concerns, many of which were similar to those highlighted during workshops held as part of the earlier feasibility study [25] (Table 2). For example, the change from metal vaginal specula in the feasibility study to single-use plastic instruments in the main trial has been associated with a reduction in rumours and concerns about the safety of genital examination associated with perceptions about the cleanliness of re-useable instruments. Blood taking remained a concern in the first year of the trial but community perceptions that blood might be collected and sold for witchcraft purposes were less common, following a variety of initiatives introduced during the feasibility study [25].

**Table 2:** List of key community concerns related to participation in the Mwanza feasibility study and MDP301 trial

**Feasibility study**		**MDP301 clinical trial**	
**Issue (ranked in order of priority)**	***Comments by workshop participants (Sep 03 -- Mar 04)***	**Issue (ranked in order of priority)**	***Comments by workshop participants (Oct 06 -- Feb 07)***

1. Blood taking	*'why do you take so much blood every time?'*	1. Allowances/reimbursal for participation	*'life is becoming expensive and allowances are not enough -- why can't they be raised?'*
	*'blood might fall into the wrong hands and be sold for witchcraft purposes'*		*'some of us have not received project T-shirts but others have already got theirs'*
			*'we are supposed to get a soda and a snack when we arrive at the clinic but sometimes we are not given'*

2. Allowances	*'we are losing money when we come to clinic'*	2. Range and quality of services provided	*'staff sometimes don't listen as much as they could'*
			*'clinics start late and are taking too long -- we spend the whole day there'*
			*'we want to bring our children when they are sick'*
			*'why can't you treat simple things like malaria?'*
			*'are the lab tests trustworthy? Why are *[HIV] *tests not in little envelopes like before?*
			*'why are some lab results not available when we come back to clinic?'*
			*'what will happen once I finish *[the trial] -- *can I still get service at the study clinic?'*

3. Speculum examinations	*'how do we know the speculum is safe *[clean]*?*'	3. Blood taking	*'they are taking too much blood -- every time two bottles'*
			*'after the blood test my heart was irregular for one week'*
			*'one time after blood was taken I had pain in my arm for three days'*

4. Range and quality of clinical services provided	*'why can't we bring our children to the clinic when they are sick?'*	4. Stigma	*'people think that the bags *[given to all participants at screening] *used to collect gel must be for ARVs and that we must be positive'*
	*'you should treat malaria and fever in children'*		*'the community is doubting that all those who join the project are HIV negative... we guess at least 40% should be positive'*
	*'our men don't like to go to hospital [for STI treatment] - why can't we bring them to the clinic'*		
	*'sometimes we wait a long time to be seen'*		
	*'some tests take a long time to come back'*		
	*'I went [to another clinic] and got my result straight away after I had already waited a long time for my result from your clinic'*		
	*'how can you help me if I am/become HIV positive?'*		

5. Stigma and confidentiality	*'people think that clinics are only for people who are HIV positive'*	5. Issues related to study gel	*'the gel increases wetness... makes men think there is some abnormality'*
	*'my photograph might appear in the newspaper with my HIV result'*		*'the project needs to provide information to men... to educate them about gel'*
			*'what will happen if gel is effective; will study volunteers get a supply?'*

Priority concerns during both the feasibility study and the main trial were primarily related to immediate issues of current clinical service provision and the need to address timeliness, efficiency and certain specific procedural matters (e.g. reimbursement levels; venepuncture) rather than broader standard of care issues. Requests to broaden the scope of services provided, in order that the children and spouses of participants could receive treatment at study clinics (rather than by referral), were raised in the feasibility study and the main trial. Access to care and support services for women living with HIV and AIDS, including antiretroviral therapy, was raised during the feasibility study workshops but appeared of less concern in the main trial, when specialist referral services had already been established for some time. Concerns about ensuring post-trial access to an effective microbicide gel were expressed only in the later series of workshops.

### Stakeholders Advisory Group recommendations

**Figure 2:**
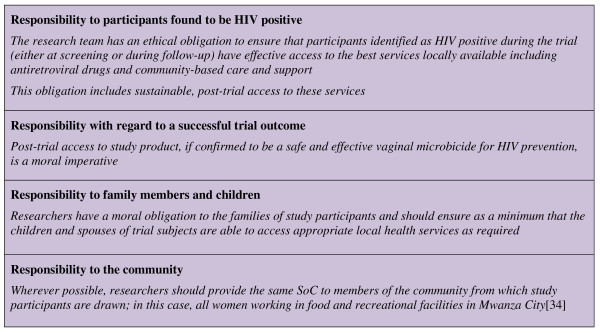
**Key recommendations of the MDP Mwanza Stakeholders Advisory Group**.

The SAG has considered SoC issues since its inception and in contrast to the community group, has focussed primarily on broader ethical issues related to clinical service provision for study participants, their families and the wider community (Figure 2). Stakeholders felt that to provide more than the best care available locally or to attempt to provide the 'worldwide best care' to trial participants would be coercive and unethical in this setting since it would not be possible to extend such services beyond those participating in the trial or to sustain such levels of care following trial cessation. In order to support local health systems and human resource capacity development, researchers were strongly advised to continue working within existing structures and to avoid setting up parallel systems wherever possible. Stakeholders also stressed concerns that client referrals from research clinics could risk stretching already overburdened local health services; and the importance of ensuring appropriate resource inputs to offset such risks. The SAG also debated concerns raised by the CAC and emphasised to the research team the critical importance of maintaining effective community engagement in the management and implementation of the trial in order to ensure that study health services continue to be delivered in a locally-appropriate, acceptable and effective manner.

### Issues and constraints in implementation

Having agreed the guiding principles for a locally-appropriate SoC package for study participants through consultation with the CAC and SAG, the research team encountered considerable difficulties successfully implementing the clinical care package (Table 1) due to a combination of logistical and socio-cultural factors, in response to which a variety of corrective actions have been taken (Table 3).

**Table 3:** Issues and constraints to SoC package implementation

**Service area and key issues**	**Comments/Action taken**
***STI services for trial participants*** Many trial participants do not receive the results of laboratory STI tests at their next scheduled visit Some participants do not receive appropriate and timely additional treatment as indicated by their STI laboratory test results	Team meetings involving clinic and data management staff suggest these issues arose due to a combination of: difficulty tracing participants in the community to ask them to return for further treatment; difficulty ensuring continuity of care between visits due to high clinic through-put and multiple clinical staff (participants not assigned designated clinician); and inadequate systems and procedures to flag new test results (so that even when participants did return to clinic and STI test results were present in their study folder, appropriate treatment was not always given) Systems for reporting STI tests were reviewed and modified Feb 08 to shorten turnaround time between testing and release of results to clinic Reporting system modified to expedite the printing of all positive laboratory results and their prompt release to clinicians resulting in targeted community follow-up e.g. participants requiring additional treatment following initial syndromic-based STI management Clinical tools developed to facilitate continuity of care training of clinicians modified to emphasize the importance of full review and continuity of care at each clinical visit

***STI services for sexual partners*** Trial participants complained that although they are being treated for STIs at study clinics they are at risk of re-infection because their sexual partners refuse to attend STI referral services A review of referral service utilisation in Jan07 (based on an internal review of trial records and referral centre notification slips) indicated poor uptake by male sexual partners (< 30%) Trial participants have continued to request that sexual partners receive treatment in study clinics rather than by referral	An internal review conducted in Jan 2007 concluded that systems for monitoring and evaluating service uptake be strengthened; that staff at the referral centre receive regular updates on the trial and training in relevant trial procedures (e.g. adverse event reporting in men); and that a greater focus be placed on male participation and support for the trial through community outreach activities. Despite implementing these initiatives, estimated service uptake remained < 30% in the year to Jul 2008. The issue of treating partners at study clinics has been explored several times in CAC and SAG meetings with the consensus view that study clinics have been established as 'a service for women provided by women' and that treating men at the same clinics would impact negatively on women's experience of clinic attendance and the trial.

***Care and support for women living with HIV*** A review of referral service utilisation in Jan07 (based on an internal review of trial records and referral centre notification slips) indicated poor uptake among women referred to the local collaborating specialist provider (< 30%)	During an internal review in Jan07, clinic staff were advised to emphasise the positive aspects of referral and to reaffirm that antiretroviral therapy can produce dramatic improvements in health and quality of life, even among those who present relatively late. Additional counselling and support were also offered to facilitate uptake but despite these measures, estimated service uptake remained < 30% in mid-2008. Informal discussions with community representatives at workshops and CAC meetings have highlighted a number of potential constraints to service uptake, including stigma, travel and opportunity costs. These issues are being investigated in the Barriers Study that will inform local and national policy on ART access among vulnerable at-risk groups.

***Gynaecology, general medical and child health *** All women with suspected cervical carcinoma and/or other gynaecological abnormalities on speculum examination have to date received specialist care in Mwanza It has not been possible to track general medical and child health service uptake. Trial participants have continued to request that children receive treatment in study clinics rather than by referral	A new Breast and Cervical Cancer Screening Clinic is being established at a tertiary care hospital in Mwanza in 2009. Plans to implement visual inspection with acetic acid (VIA) in Dar es Salaam and Mwanza are on-going (*K Shapiro, personal communication*) Better systems for tracking service uptake following referral are required in future clinical trials in Mwanza. CAC and SAG meetings have discussed service provision for children periodically during the trial but on each occasion have accepted that a combination of logistical, funding, human resource, sustainability and other issues make it difficult for the MDP301 Mwanza trial team to provide child health services directly via study clinics. All parties have agreed that this issue be carefully assessed in future trial design.

Despite these significant contextual factors, progress has been made in meeting the four SAG recommendations. The project has established collaborative agreements with local service providers, which have secured funding through government and external donors in the medium-to-long term ensuring treatment and care services for women living with HIV and AIDS can be sustained beyond the end of the MDP301 trial in Mwanza. To ensure post-trial access to a successful vaginal microbicide, a register of trial participants is being collated that will facilitate community tracing and subject enrolment into future named-client phase IV trials. Referral services have been established for sexual partners of study participants and procedures instituted to monitor STI service uptake and to consolidate partnership working with designated specialist providers in Mwanza, but there has been less success in child health services (Table 3). Finally, the project has made a commitment to provide services to women working in food and recreational facilities in Mwanza, irrespective of whether they are currently trial participants, but does ask they have at least attended a trial screening visit or been involved in the earlier feasibility or pilot studies.

### Case studies

Another area in which the research team, community representatives and stakeholders experienced difficulties was in trying to decide where researchers' responsibility and obligation to individual participants ended. The following case study illustrates some of the ethical dilemmas faced.

#### Case study: Acute psychosis and multiple vitamin deficiencies secondary to alcoholism

**Figure 3:**
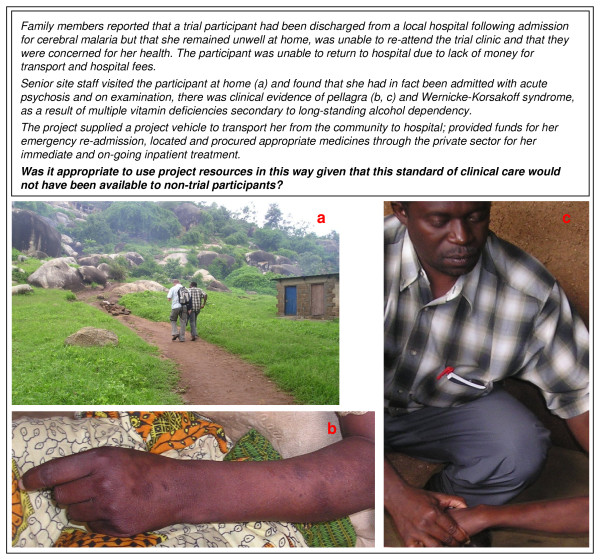
**Case study: Acute psychosis and multiple vitamin deficiencies secondary to alcoholism**.

Researchers involved in the management of this case (Figure 3) felt that they had a clear duty of care to this participant and that it was appropriate to use whatever project resources were required to avert a life threatening situation. Ethically and morally we believe it would be difficult to argue against this position. There was, however, some debate within the research team as to whether our actions could be viewed as inappropriate in the context of our working definition of SoC based on access to the best locally-available care, since the level of assistance provided was clearly in excess of that available to members of the community outside the study population i.e. women who were not working in eligible food or recreational facilities in Mwanza. Having become involved in this patient's care, it was also unclear at what point and how we would decide whether or not to continue our assistance, including financial support. For example, would it still be appropriate for the project to pay for the costs of referral to a regional medical centre (e.g. in Dar es Salaam or Nairobi) should the patient's condition indicate that this was necessary? Finally, some members of the team were concerned that our actions could be misunderstood in the community as being part of some sort of 'cover up' to remove a woman experiencing severe side effects due to her participation in the trial from further public scrutiny. Consultation with community representatives and members of the CAC and SAG subsequent to these events revealed that the opposite was in fact the case: the prevailing view in the community was that the project 'truly cared about the women' participating in the trial and was prepared to go to great lengths to assist them should they encounter problems, even if these problems appeared unrelated to trial participation.

#### Case study: Vaginal bleeding due to ectopic pregnancy

In another example, a woman with heavy vaginal bleeding received specialist medical care and confirmation of a provisional diagnosis of ectopic pregnancy within hours of presentation at an MDP Mwanza trial clinic following the urgent intervention of the research team. Researchers again felt they had a clear moral obligation to this participant and that it was appropriate to mobilise all resources necessary (including staff time; project vehicles; specialist referral, diagnostic, treatment and in-patient costs) to ensure access to the best possible care available locally, even if this would have been impossible for her were she not enrolled in the MDP301 trial.

## Discussion

Participatory methodologies enabled effective partnerships between researchers, participant representatives and community stakeholders to be developed during a vaginal microbicide trial in Mwanza, Tanzania and facilitated open dialogue and consensus on what constitutes an appropriate standard of clinical care in this setting. By actively seeking input from a combination of primary and secondary stakeholders we were able to capture a broad range of information from the practical and highly-specific (advising how study clinics might be more effectively and appropriately implemented) to the conceptual (researchers' moral and ethical obligations to trial participants, their families and the broader community).

The Mwanza experience reflects that of researchers and ethics committees in other resource-limited settings where locally-available levels of health care have been taken into account when determining what constitutes appropriate SoC rather than attempting to provide the highest possible SoC as advocated in the Declaration of Helsinki [1,2]. For example, a recent systematic review of 73 clinical trials of HIV prevention and treatment (n = 34); malaria prevention (n = 29); and tuberculosis treatment (n = 13), all conducted in sub-Saharan Africa, found that only 16% provided therapy consistent with the 'best current' standards of clinical care based on internationally agreed guidelines, such as those produced by the International AIDS Society or the World Health Organization [15].

**Figure 4:**
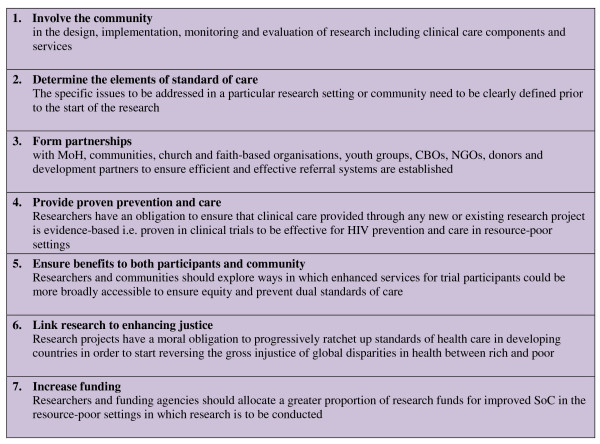
**Seven-Step Framework towards improved Standards of Care (adapted from Shapiro & Benatar, 2005 **[16]).

Our approach closely follows that advocated by Shapiro and Benatar, who describe a seven step framework (Figure 4) to guide improved standards of care, based upon the core principles of equity, social justice and beneficence [16].

How well does our research measure up to this framework? The MDP301 Trial in Mwanza has been conducted in a specific occupational group within a defined geographical area among women vulnerable to HIV/STI infection, social harm and stigmatisation[25,27]. By actively involving women who consider themselves part of a community defined by occupation, geography and research participation; by establishing collaborative partnerships within the broader local community; by defining key elements of SoC; and by providing evidence-based interventions (such as STI syndromic management; VCT and risk reduction counselling) a locally-appropriate care package was developed. The feasibility study, pilot study and clinical trial have all been implemented through community-based clinics allowing women who are typically marginalised within the broader Mwanza community, and who thereby find it difficult to access basic health services, to receive high-quality, evidence-based care. In such settings, we believe it is a moral imperative to attempt to close gaps in health care within communities and that researchers should actively seek to 'ratchet up' the standard of care available to the most vulnerable members of society[16,24]. The MDP and wider Mwanza research group have attempted to link research and justice by investigating options for the introduction of cervical cancer screening and colposcopy at population-level in Mwanza and by facilitating specialist training in genitourinary and HIV medicine to consolidate local quality health care provision. Finally, a significant proportion of the MDP Mwanza budget is already allocated to health service provision in order to establish and maintain an entirely new community-based health service.

Despite an approach based on the Seven-Step Framework, the realities of trial implementation in a complex resource-poor setting meant that several key SoC elements were implemented with only partial success, most importantly adequate provision of care for women found to be HIV seropositive during the trial and STI treatment services for male sexual partners. A recent review by the Global Campaign for Microbicides (GCM), which mapped SoC at microbicide trial sites in Benin, Ghana, Nigeria, South Africa, Tanzania and Zimbabwe[12], found such difficulties to be common across all African trial sites and urged those funding, sponsoring and coordinating HIV prevention research to continue striving to 'ratchet up' local standards of care in communities hosting clinical trials. Key recommendations of the review include the need for a greater emphasis on partnership working with communities and local health service providers (co-location, skills sharing and capacity building); use of referrals as a mechanism for care; identification and reduction of barriers to accessing care; and greater communication and information sharing within the HIV prevention research field to highlight approaches that appear to work and those that do not. The limited resources available for clinical trials in developing countries mean that community stakeholders, researchers and sponsors will continue to face extremely difficult choices when attempting to reconcile the immediate needs of trial participants with the potential for longer term benefit to the wider community.

Can community-level and individual SoC considerations be reconciled in HIV prevention research? Deciding the extent and limits of researchers' involvement in the management of complex medical problems and emergencies arising in settings such as Mwanza is problematic. Our obligation and duty of care to participants has been tested by a number of specific case studies, which suggest that in addition to developing and agreeing broad SoC guidelines and principles in any given developing country research environment there will always be a need to make case-by-case decisions. We suggest that by establishing effective, open and trusting partnerships with local communities and stakeholders, researchers will be better equipped to make informed choices in difficult circumstances and to take decisions that make sense in the specific context in which they work. Whilst individual high-profile clinical cases are important, it is vital that researchers monitor trial participants' access to and uptake of routine, non-emergency health services and consider operational research to clarify potential barriers and constraints in order to inform future research program implementation.

## Conclusion

Participatory methodologies enabled effective partnerships between researchers, participant representatives and community stakeholders to be developed in Mwanza, Tanzania and facilitated local dialogue and consensus on what constitutes a locally-appropriate standard of care in the context of a phase III vaginal microbicide trial in this setting.

## Competing interests

The authors declare that they have no competing interests.

## Authors' contributions

All authors contributed to the development and revision of draft manuscripts, read and approved the final version of the manuscript. AV, CS, and SL designed and coordinated participatory research and community liaison activities, coordinated and managed fieldwork, data collection, analysis and interpretation with support and guidance from JC, SMc and RH. All authors contributed to the design and development of locally-specific standard of care guidelines. AV conducted the literature review, drafted the manuscript and incorporated revisions into the final version for publication in an iterative process involving input from all co-authors.

## Pre-publication history

The pre-publication history for this paper can be accessed here:


